# The New Radiant Energy

**Published:** 1896-02-08

**Authors:** 


### The New Radiant Energy.

One of the penalties of publicity, so far as regards new discoveries, arises from the extraordinary avidity with which all sorts and conditions of men seize upon bald facts and talk as if they knew all about them—for a journalist is nothing if not omniscient. It may be as well, then, to draw attention to how little we as yet know about that manifestation of energy which some journals, with a happy knack of misdescription, speak of as the new photography. That some invisible form of energy radiates from a vacuum tube through which an electric discharge is passing is plain enough from its effects upon a sensitive plate exposed to its rays; but that it is not light and is not heat, in the rough sense of the term, is equally plain. It is not perceptible to our senses; it is stopped by substances which to the better-known forms of radiation are transparent, and passes readily through others which are to them opaque. This rough distinction, however, would not be enough to separate it from ordinary light in scientific parlance, for it is well known that there are rays, extending far beyond the spectrum at both ends, which, while completely invisible, conform to all the other laws by which the propagation of light is ruled, and thus may be properly looked upon as rays of light which are not seen only because our eyes are incapable of seeing them. The exact analogue of this is to be found in regard to sound. The ear is but an imperfect instrument, the range of the vibrations it can hear varying in different individuals much as does the range of the vibrations which different voices can produce. Some people can walk in the still night quite oblivious of the shrill screaming of the bat, others are deaf to deep-toned organ notes, although they feel them through their boots. But these inaudible vibrations are none the less to be spoken of as sound because the individual ear does not perceive them, and so the ultra-red and ultra-violet rays although invisible, must, from their relation to the other portions of the spectrum, be classed as light. But the new form of radiation is not either ultra-red or ultra-violet. It cares nothing for either lenses or prisms, it is unrefracted and goes straight through. Here, then, comes in our ignorance, and here also comes in the real importance of the discovery so far as it has gone. If this is light, some of our notions as to light vibrations must be modified; if it is not light, what is it? People have been so interested in what they call the practical applications of the discovery that many have failed to see that its real importance lies in its bearing upon theory. No doubt there is something mysterious and attractive in photographing the invisible, and demonstrating the presence of keys in boxes and bullets in patients' hands, but the importance of such performances is trifling compared with the scientific issues which would be at stake were it to turn out that some modification should be necessary in the theories on the truth of which so much of our boasted knowledge hinges.

Beyond the fact that these rays exist our knowledge of them seems to be chiefly negative. They are not susceptible of refraction or diffraction, or concentration by a lens, or reflection by ordinary reflecting surfaces, and, more interesting still, they do not seem to be capable of being polarised.

All this appears to separate them from those transversal vibrations, be they of position or of stress, by which we are accustomed to explain the propagation of light. Yet that they are in touch with the more ordinary forms of radiant energy is shown by the fact that certain objects by which they may be received are capable of converting them into the more ordinary forms of force, in the one case into fluorescence (which in fact is light), and into the other into chemical change exactly analogous to that which is produced by ordinary actinic rays. As we said before, here comes in our ignorance. We do not know for certain that, accompanying the vibrations of light which are transverse to the line of propagation, there may not be others which, taking place in the longitudinal direction, might behave much as do these newly-discovered rays; on the other hand, we do not yet know how infinitely short transverse vibrations might act, or that they would refract, reflect, and polarise, in the way that ordinary light undulations do; and it would appear that it is as a means of solving these obscure questions that the new rays are of real importance, and not merely as a means of illuminating dark areas in the body and seeing keys through planks of wood.

